# Non‐osseous intradural tuberculoma of the thoracic spine with compressive myelopathy

**DOI:** 10.1002/ccr3.8131

**Published:** 2023-11-02

**Authors:** Shyam Duvuru, Vivek Sanker, Syed Naureen, Gupta Prakash, Rajurkar Sanjana, Tirth Dave

**Affiliations:** ^1^ Department of Neurosurgery Apollo Specialty Hospitals Madurai Tamil Nadu India; ^2^ Team Erevnites Trivandrum India; ^3^ Research Assistant, Department of Neurosurgery Trivandrum Medical College Kerala India; ^4^ UT MD Anderson Cancer Center Houston Texas USA; ^5^ Virgen Milagrosa University Foundation College of Medicine San Carlos City Philippines; ^6^ Datta Meghe Institute of Higher Education and Research JNMC Wardha India; ^7^ Bukovinian State Medical University Chernivtsi Ukraine

**Keywords:** myelopathy, spinal cord, thoracic spine, tuberculoma

## Abstract

**Key Clinical Message:**

An uncommon form of CNS tuberculosis called non‐osseous IDEM tuberculoma frequently results from paradoxical drug interactions. It should be considered one of the differentials when patients receiving ATT experience acute neurological impairment.

**Abstract:**

Tuberculoma affecting the spinal cord is a rare condition in modern times. The occurrence of non‐osseous intradural tuberculosis, specifically in the spine, is even more exceptional. In fact, it is uncommon to encounter an intradural extramedullary tuberculous granuloma that lacks radiological indications of vertebral involvement, especially within the thoracic region. We present a case of a patient with a neurological deficit caused by a non‐osseous intradural tuberculoma in the thoracic region, without any associated bone involvement. The patient experienced a gradual deterioration of neurological function. An MRI of the thoracic spine revealed the presence of a tuberculoma located intradurally, extramedullary, and juxtamedullary of the T5 vertebra. The compression of the spinal cord resulted in paraparesis which was worsening to paraplegia. A D4–D6 laminectomy and microsurgical excision were performed under intraoperative neurophysiological monitoring (IONM), and the patient showed clinical recovery. Excellent clinical outcomes were achieved. However, it is crucial to consider the possibility of a non‐osseous intradural tuberculoma as a rare condition when encountering a SOL, particularly in patients with a history of tuberculosis and spinal cord compression. In cases where a progressing neurological deficit is present, a combination of surgical intervention and anti‐tuberculous treatment should be considered as the optimal approach.

## Graphical abstract

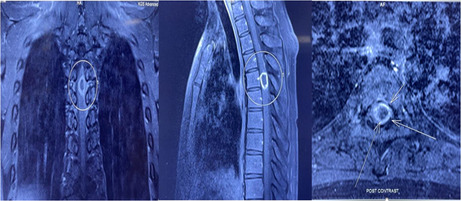

## 1 INTRODUCTION

Tuberculosis remains a significant global health burden, with central nervous system (CNS) involvement being a serious manifestation. The CNS involvement in developing countries constitutes nearly 10% of all tuberculosis patients.^1^ In the differential diagnosis of extensive spinal cord injuries, particularly in young patients with a history of pulmonary tuberculosis or tuberculous meningitis, it is important to consider the possibility of an intradural extramedullary tuberculoma.^2^

Here we will be discussing a case of intradural extramedullary tuberculosis of the thoracic spine, with progressive neurological dysfunction. Surgery aims to decompress the spinal cord and remove the tuberculoma, thereby relieving the pressure on the neural tissues and preventing further neurological deterioration.^3^, ^4^, ^5^ The specific surgical technique employed depends on the location and extent of the tuberculoma.

Following surgery, anti‐tubercular therapy is initiated to target the underlying tuberculosis infection. This typically involves a combination of multiple anti‐tuberculosis medications, such as isoniazid, rifampicin, ethambutol, and pyrazinamide.^2^ The duration of anti‐tubercular therapy may vary but generally lasts several months to ensure complete eradication of the infection. Concurrently, physiotherapy and rehabilitation assume significance in facilitating the functional restoration and enhancing the overall quality of life.^1^

This report aims to discuss the diagnostic challenges, treatment strategy, and clinical outcomes, highlighting the importance of a multidisciplinary approach in managing this uncommon condition.

## 2 CASE REPORT

A 29‐year‐old man, who had been under treatment for disseminated tuberculosis for 7 months, was admitted with progressive weakness of both lower limbs leading to difficulty in walking. There was no history of fever, cough, palpitations, or breathlessness. Physical examination revealed a palpable spleen but no lymphadenopathy. He had spastic paraparesis with involvement in right side more than the left. There was severe girdle pain at T5 level. The patient underwent a computed tomography (CT) chest, which revealed multiple patchy resolving ground glass opacities and interlobular septal thickening in bilateral lower lobes, predominantly superior and posterior basal segments, suggesting resolving tuberculosis. Also, mild centrilobular emphysema in bilateral upper lobes with sub‐centimeter right paratracheal and left paraaortic lymph nodes, the largest measuring 7 mm, were present. Other findings included splenomegaly measuring 17.6 cm and calcified hepatic granulomas (segment VII).

**FIGURE 1:**
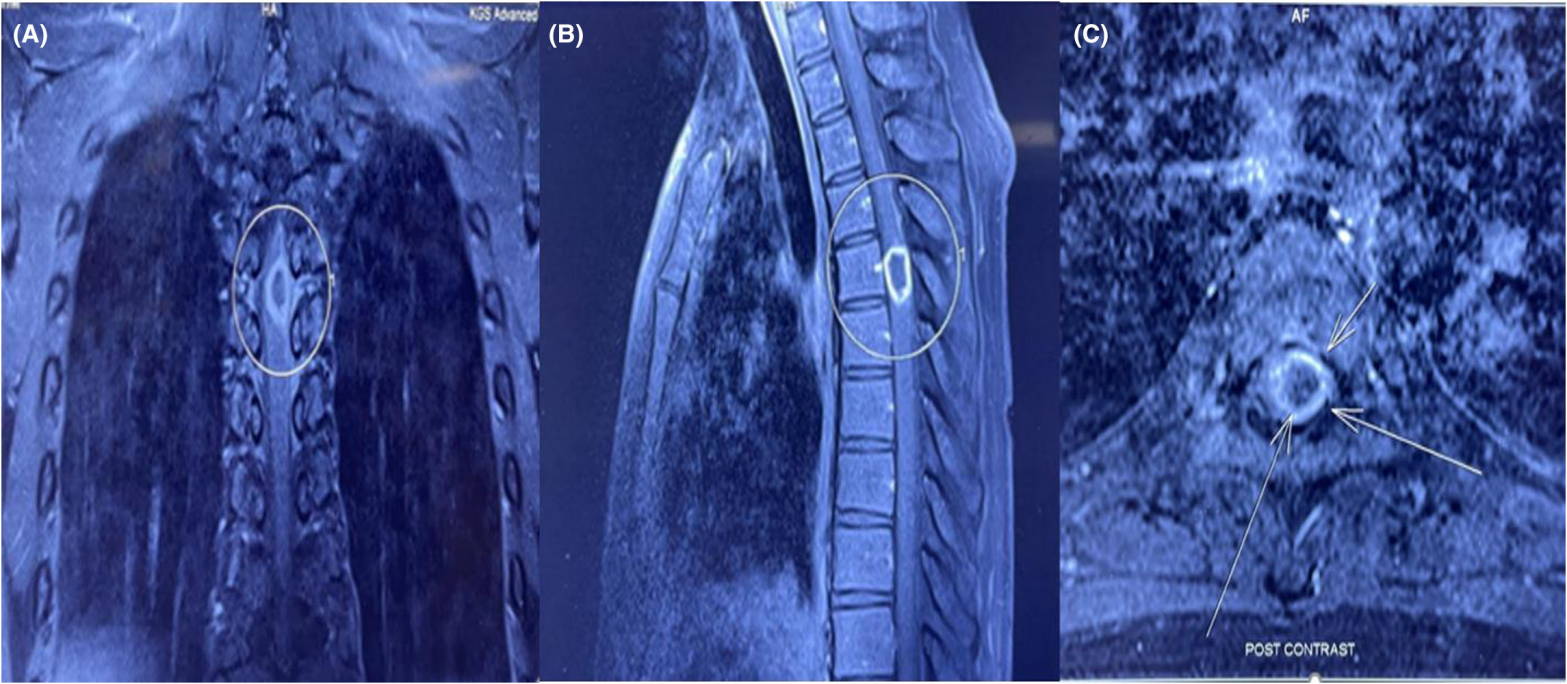
The MRI images of the thoracic spine. (A) Coronal T1‐weighted MRI image shows a ring‐like enhancing intradural lesion after gadolinium. (B) Sagittal T1‐weighted MRI image shows a 2.1 × 1.7 × 1.45 cm intradural/juxtamedullary and extramedullary mass with central necrosis and marginal enhancement after gadolinium suggestive of immature tuberculoma at T5 level. (C) Axial T2‐weighted image.

Magnetic Resonance Imaging (MRI) thoracic spine revealed a single 2.1 × 1.7 × 1.45 cm intradural/juxtamedullary and extramedullary mass appearing as well‐defined T1‐weighted low signal/T2‐weighted FLAIR bright signal with central necrosis and marginal enhancement after gadolinium (Gd) suggestive of immature tuberculoma at T5 level with surrounding edema (Figure 1A–C).

**FIGURE 2:**
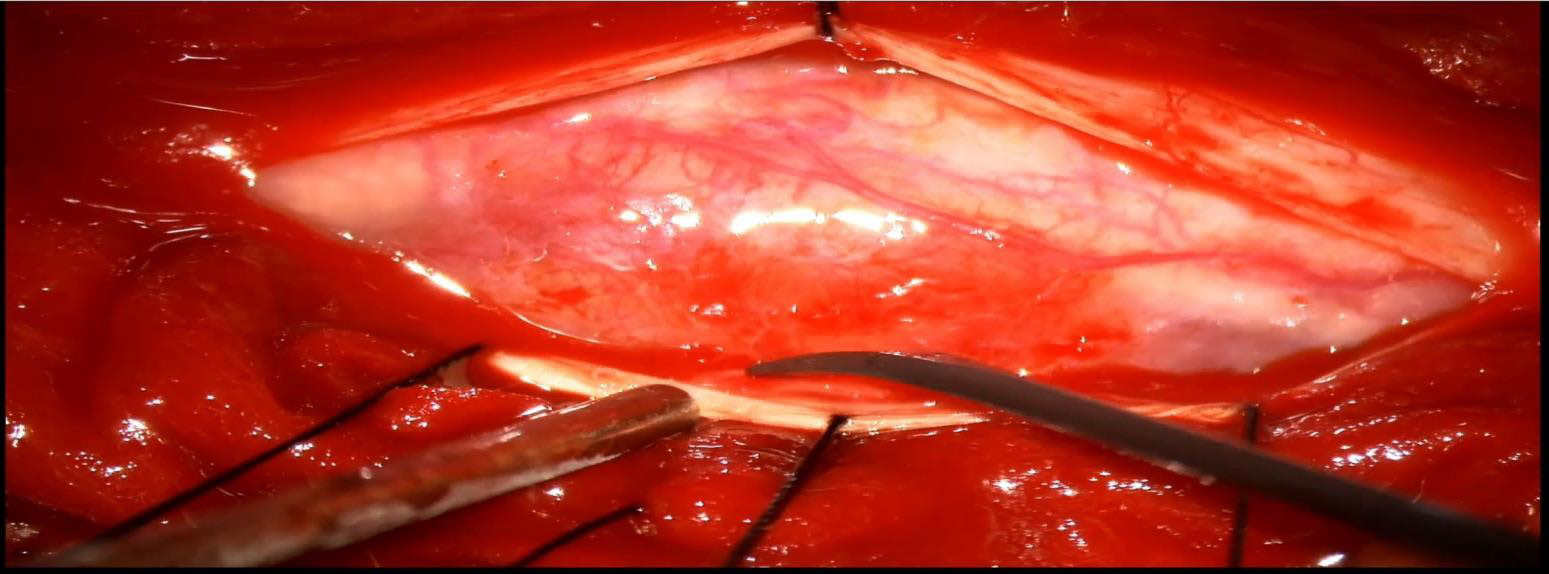
The capsule and necrotic component is seen which is densely adherent to spinal cord.

No evidence of vertebral TB, spinal tract infiltration, or engulfment was noted. A diagnosis of D5 intradural extramedullary space‐occupying lesion (SOL) with spastic paresis, cord compression, compressive myelopathy, disseminated tuberculosis, and post‐tuberculous medication‐induced hepatic granuloma was made. D4‐D6 laminectomy and microsurgical excision under IONM were planned to remove the lesion. After pre‐anesthetic assessment and consent, the patient underwent the procedure with the head held in a horseshoe, subperiosteal muscle dissection made, and laminae exposed. Durotomy was performed under a microscope, and the lesion was visualized. A grayish‐white, firm lesion with a central necrotic area was exposed (Figure 2).

**FIGURE 3:**
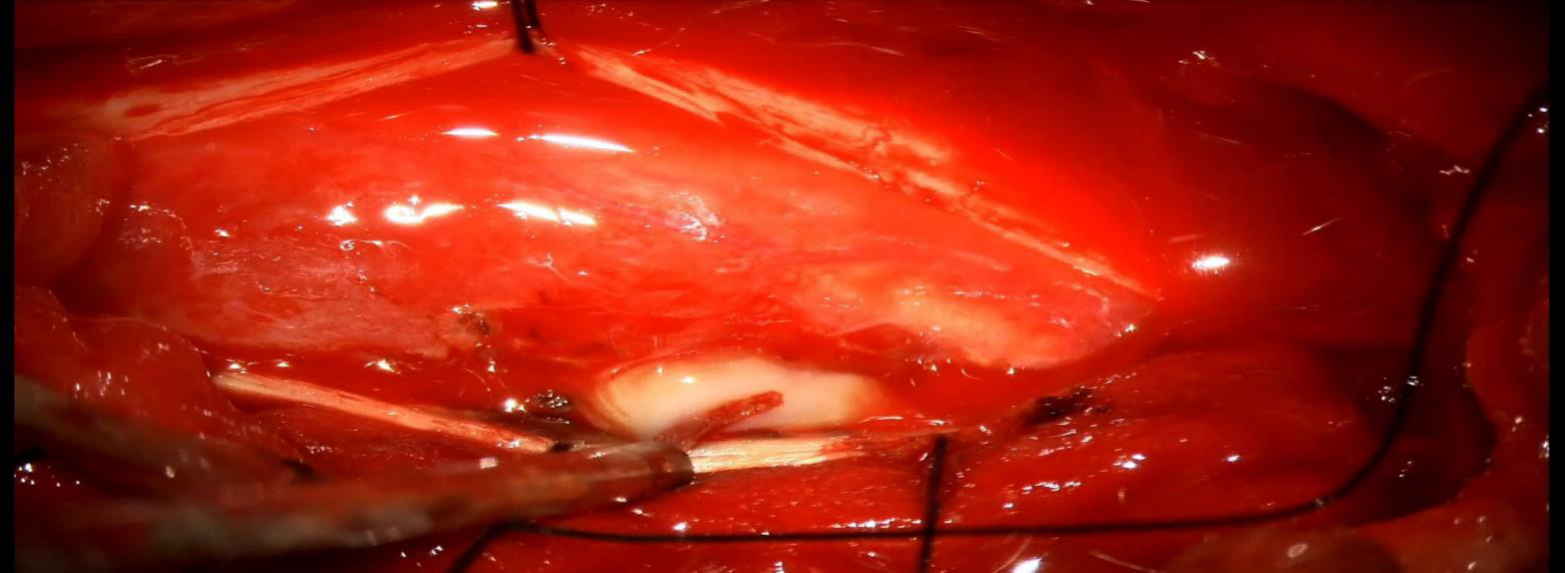
Intraoperative picture showing a densely adherent lesion with neovascularization.

**FIGURE 4:**
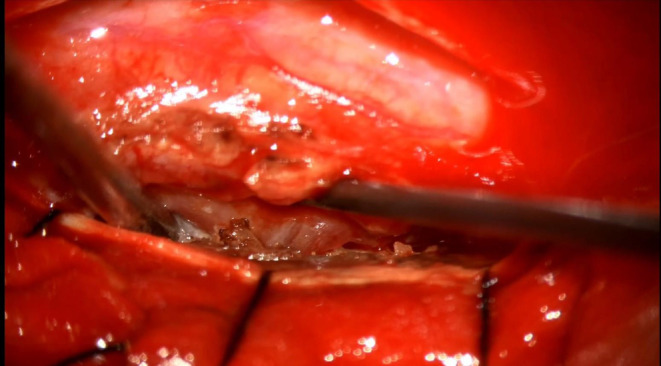
The capsule is incised, and pus is delivered off the lesion.

**FIGURE 5:**
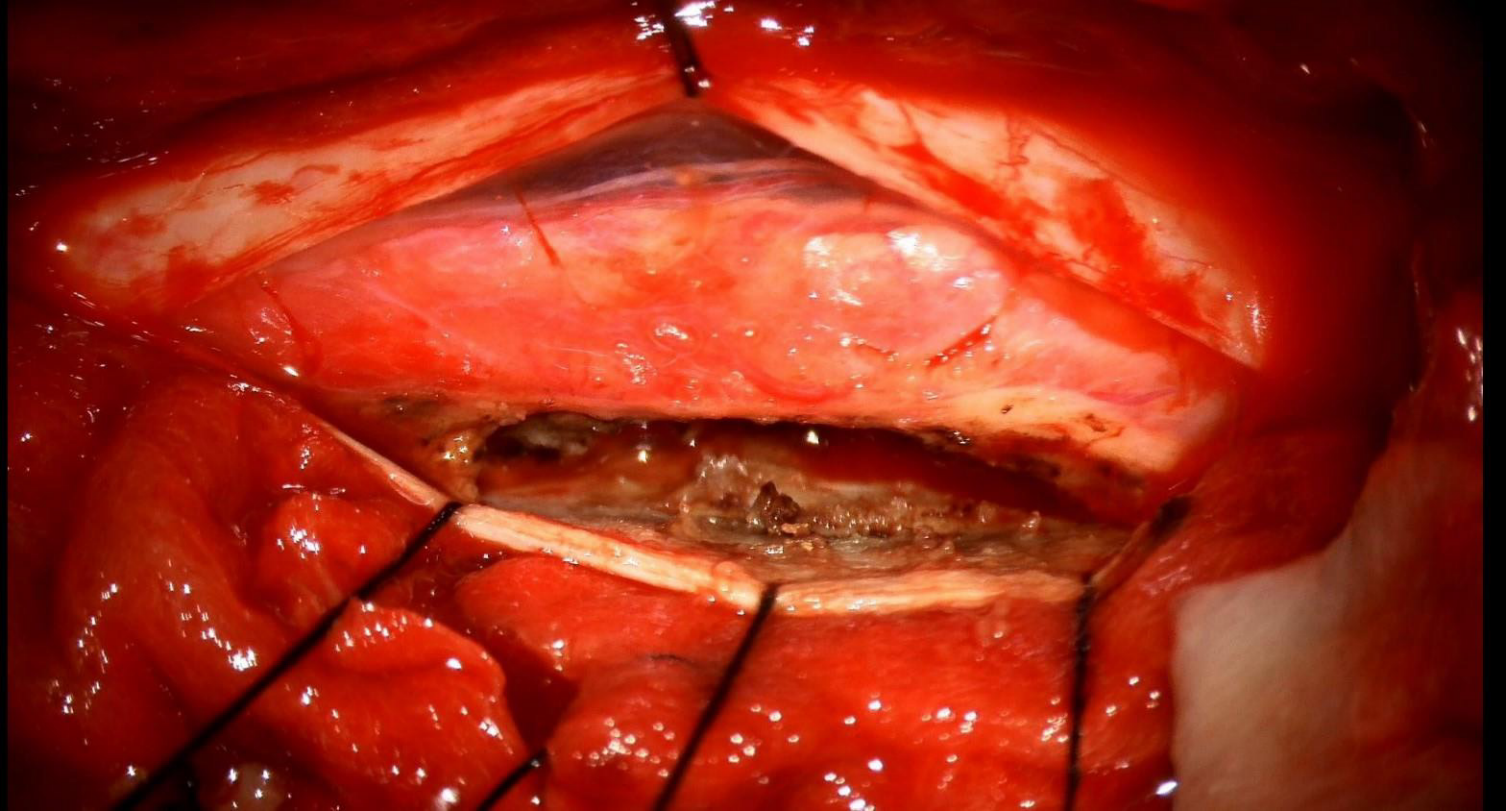
Post‐excision status with decompression of the spinal cord.

The pus was aspirated, followed by internal bulking using ultrasonic surgical aspirator (Figures 3, 4, 5). The thin rim of the capsule was left behind but was coagulated well. Homeostasis was achieved, and the dura was closed using a 5–0 proline continuous suture. The patient tolerated the procedure well. After surgery, he was treated with antibiotics, anti‐diabetics, steroids, anti‐tubercular drugs, anti‐inflammatory, gastroprotectives, antiemetics, and other supportive measures. Physiotherapy measures were instituted. The neurologist was involved in his paraparesis care, and an endocrinologist for glycemic control. He gradually improved symptomatically, able to walk with minimal support.

## 3 DISCUSSION

Tuberculosis (TB) remains a common health issue in developing countries, even though TB has not been seen widely as before.^6^ Osseous TB involvement may account for up to 35% of patients with extrapulmonary TB, and vertebral osteomyelitis infections (Pott's disease) account for 25%–60% of all osseous infections caused by TB.^7^, ^8^, ^9^ Approximately 10% of patients with extrapulmonary tuberculosis experience CNS involvement, typically resulting from the bacteremic stage of the disease. During this stage, tuberculous lesions known as Rich foci may develop in the meninges or within the brain or spinal cord tissue.^10^, ^11^ After several months or even years, these foci can rupture into the cerebrospinal fluid, leading to meningitis, or they can enlarge and form tuberculomas within the brain or spinal cord parenchyma.

The pathogenesis of central nervous system tuberculosis involves the development of small foci of tuberculosis, known as rich foci, in the brain, spinal cord, or meninges.^10^ Earlier studies by Rich and McCordock showed that TBM requires direct inoculation of the bacilli through a meningeal focus in the central nervous system. However, later studies showed that disseminated tuberculosis plays a crucial role in the development of tuberculosis.^12^ Tumor necrosis factor alpha (TNF‐α) is a critical cytokine in *M. tuberculosis* neuropathogenesis. Although it helps in granuloma formation and control of mycobacterial infections, local production of TNF‐α in the central nervous system can alter the permeability of the blood–brain barrier and promote the progression of TBM.^13^

Human microglia are selectively infected by *M. tuberculosis*, and the CD14 receptor facilitates engulfment of unopsonized bacilli by microglia. Microglia infected with *M. tuberculosis* produce a variety of cytokines and chemokines, including TNF‐α, IL‐6, IL‐1β, CCL2, CCL5, and CXCL10. Microglia play a central role in the neuropathogenesis of tuberculosis, and their infection can lead to immunosuppressive effects, especially in more virulent strains.^14^

Similarly, among various forms of spinal TB involvement, such as Pott disease, non‐osseous spinal TB, tuberculous spinal meningitis, and tuberculous arachnoiditis, non‐osseous spinal tuberculoma is infrequent. In a review conducted by Dastur, 74 cases of spinal tuberculomas were analyzed, revealing that 65% were located extradurally, 8% were intramedullary, 5% were intradural extramedullary, and 20% were arachnoidal.^15^, ^16^, ^17^ Non‐osseous spinal tuberculomas typically originate from a primary pulmonary focus, spreading hematogenously or through direct extension from hilar lymph nodes.^18^ In our case, a rare and unexpected involvement occurred in the form of a non‐osseous IDEM tuberculoma of the spinal cord, resulting in paraparesis despite the patient receiving anti‐tubercular therapy (ATT). To our knowledge, this is the first reported case of a tuberculoma at the juxtamedullary location in the thoracic region.

The clinical presentation of the case aligns with the findings reported in the literature, which often show a higher prevalence among males.^19^ The most common presentation in our case was spastic paraplegia, post‐ATT liver failure, and respiratory problems associated with tuberculosis and COVID‐19. Nevertheless, tuberculomas are slow growing with areas of necrosis that are encapsulated, avascular, and infrequently calcification. Our patient exhibited involvement of the thoracic spinal cord, which aligns with the reported incidence of up to 70% of cases in the literature. The thoracic spine is the most frequently affected site in Pott's syndrome.^20^ The radiological imaging findings of tuberculomas can vary depending on the stage of the lesion. On MRI, tuberculomas may present with noncaseating granulomas or caseating granulomas characterized by a solid or liquid center.^21^ The tuberculous lesion typically appears isointense on T1W images, isointense to hypointense on T2Wimages, and exhibits ring enhancement with a hypointense center on gadolinium‐enhanced MR scans. As the lesion undergoes caseation, the center becomes bright and gives rise to a target sign, as observed in our case.^22^, ^23^, ^24^

A combination of surgical intervention and medical treatment has shown excellent outcomes. While some authors argue that anti‐tuberculous therapy alone is sufficient when a paradoxical reaction develops into a tuberculoma,^25^, ^26^ a literature review on intradural extramedullary spinal tuberculoma confirms the limited effectiveness of medical therapy alone and highlights the need for surgical intervention.^27^, ^28^, ^29^, ^30^, ^31^, ^32^, ^33^ The reason for the poor response to chemotherapy in this condition remains unclear. Therefore, we believe that surgery is warranted when intradural extramedullary spinal tuberculoma arises as a paradoxical response to therapy.

The list of similar published cases is mentioned in Table 1.

**TABLE 1:** List of case reports having similar presentation.

Case report	Case age/Sex	Symptoms	Radiographic findings	Treatment plan	Outcome
Kim et al. 2000^34^	49/F	Seizure, facial palsy, left upper extremity weakness, paraparesis, hypesthesia below the T6 dermatome, urine incontinence	IDEM mass between the T1 and T2 spinal levels	Surgery and anti‐tuberculous drugs	Paraparesis has improved, and urine function is normal
Muthukumar et al. 2006^35^	14/F	Tonic–clonic seizure	Lesion in the spinal cord	T7–T12 Laminectomy	Power gradually increased during a 3‐month period
	45/F	Numbness, sphincter problems, and lower‐extremity inability	T1‐weighted scans show an isointense lesion spreading the cord, whereas T2‐weighted images show hypointensity with a central zregion of hyperintensity	T2–T5 Laminectomy	The patient's neurological condition had not improved
Hristea et al. 2008^36^	20/M	Lower‐limb weakness and disorientation	Obliterated thoracic and lumbar subarachnoid space	IV dexamethasone and intrathecal methylprednisolone	when supported, walk
	40/M	Right‐leg paresthesia, walking difficulties, and bladder incontinence	Multiple intramedullary lesions at D4, D5–6, D8, D10, and D11	IV dexamethasone and intrathecal methylprednisolone	The patient was able to walk without assistance, had modest sensory loss, moderate spasticity, and no sphincter problems
	29/M	Sensory loss, paresthesia, gradual lower‐limb weakness, and walking difficulties	At T8‐9, a solitary intramedullary mass suggests tuberculoma	IV dexamethasone and intrathecal methylprednisolone	The patient was able to walk on his own
Ding et al. 2012^37^	63/M	Weak hands and arms neck and thoracic discomfort	A shuttle‐like mass with low signal intensity extending from C2 to C4 in the posterior epidural region, an intraspinal extradural mass, and a compressed and edematous spinal cord	C2‐C4 laminectomy was used to decompress the spinal cord and remove the tumor	The patient recovered completely neurologically
Verma et al. 2013^38^	68/M	Low back discomfort, left‐sided lower‐extremity weakness, and radiculopathy	A lobulated enhancing intrathecal mass at L1	Bilateral laminectomy and medial facetectomy	Reported minor discomfort, independent walking, and persistent weakness in just the left tibialis anterior muscle
Venkatesh et al. 2018^39^	31/M	Mid‐backache with growing weakening of both lower limbs, urgent micturition	T1 hypointense and T2 heterointense lesion seen posterior to the thoracic spinal cord, ranging from C7 to D5 vertebral levels	D1–D5 laminectomy	The patient showed normal bladder function and increased strength in both lower limbs (Grade 4–5)

## 4 CONCLUSIONS

IDEM tuberculoma is a rare manifestation of CNS tuberculosis, often arising as a result of paradoxical reactions to anti‐tubercular medication. When patients on anti‐tubercular therapy develop new‐onset neurological deficits indicative of compressive myelopathy, IDEM tuberculoma should be considered as one of the potential causes. Early and accurate diagnosis, along with timely initiation of appropriate medical or surgical interventions, is crucial to prevent irreversible dysfunction. Excision plays a pivotal role in the treatment of this uncommon condition.

## AUTHOR CONTRIBUTIONS

**Shyam Duvuru:** Conceptualization; data curation; formal analysis; investigation; methodology; writing – original draft; writing – review and editing. **Vivek Sanker:** Formal analysis; investigation; methodology; project administration; resources; supervision; validation; visualization; writing – original draft; writing – review and editing. **Syed Naureen:** Resources; software; supervision; validation; visualization; writing – original draft; writing – review and editing. **Gupta Prakash:** Software; supervision; validation; visualization; writing – original draft; writing – review and editing. **Rajurkar Sanjana:** Resources; software; supervision; validation; visualization; writing – original draft; writing – review and editing. **Tirth Dave:** Resources; software; supervision; validation; visualization; writing – original draft; writing – review and editing.

## FUNDING INFORMATION

None.

## CONFLICT OF INTEREST STATEMENT

None declared.

## ETHICS STATEMENT

The ethical approval was not required for the case report as per the country's guidelines.

## CONSENT

Written informed consent was obtained from the patient to publish this report.

## Data Availability

The data that support the findings of this study are available on request from the corresponding author. The data are not publicly available due to privacy or ethical restrictions.
